# Relationship of Preceptor Ad-hoc Entrustment Decisions to Students’ Clinical Skills Performance

**DOI:** 10.15694/mep.2020.000061.1

**Published:** 2020-04-02

**Authors:** Pamela Basehore, Shiyuan Wang, Mehwish Khan

**Affiliations:** 1 Rowan University School of Osteopathic Medicine

**Keywords:** Entrustable professional activities, Undergraduate medical education, Clinical skills, Competencies, workplace-based assessment

## Abstract

This article was migrated. The article was marked as recommended.

Using a workplace evaluation instrument that included clinical skills evaluation and supervisory EPA scale, this study provided empirical evidence of the relationship between preceptor evaluation of student clinical ability and their entrustment decisions at the medical student level, and advanced the understanding of general conditions that ad-hoc entrustment decision are based upon. A total of 4217 evaluations from 353 third-year medical students were included in the study. The analyses focused on entrustment decisions for seven EPAs (EPA1, 2, 3, 5, 6, 7, and 9) and the relationship with related clinical skill (e.g. history taking) performance. Pearson’s correlations showed statistically significant and positive correlations within clerkships (r= 0.43-0.75) and across clerkships (r= 0.70-0.77). Analyses of individual entrustment level and skill rating also revealed that the lowest level of entrustment was predominantly associated with the rating of *developing* on corresponding skills, whereas the highest level of entrustment with the rating of *approaching advanced*/ *advanced*; the link between the three middle entrustment levels and particular skill ratings were less salient. Overall, these patterns of association between individual entrustment level and skill rating varied by EPAs as well as level of entrustment. Limitations of the current study were also discussed.

## Introduction

Outcome-based assessment in the clinical setting is an important ingredient of competency-based medical education and is required to meaningfully evaluate a learner’s progression to competence ( Frank *et al.*, 2010; Hawkins *et al.*, 2015). The optimized context and method of assessing such competencies is in an authentic workplace setting where experienced clinicians make judgments about a learner’s performance ( Gruppen *et al.*, 2018). Workplace-based assessment (WBA) is known to provide effective insight on a learner’s true ability, but can also reflect preceptor bias ( Yeates *et al.*, 2013) or inconsistencies in ratings despite general agreement among raters on trainee performance ( Crossley *et al.*, 2011). The subjectivity of clinical ratings is a challenge that has been difficult to overcome despite efforts to create more objective measures ( Hodges, 2013) and precise rating scales ( Driessen and Scheele, 2013).

In recent years, entrustment scales, defined as “behaviorally anchored ordinal scales based upon progression to competence” ( Rekman *et al*., 2016a, p. 186), were introduced as more clinically meaningful and useful for assessors in the practice setting ( Rekman *et al*., 2016a). The act of entrustment is inherent in medical training where supervisors make day-to-day decisions to allow leaners to perform professional activities with or without supervision ( ten Cate, Snell and Carraccio, 2010; Gingerich, 2015). Entrustment decisions blend the typical assessment of clinical performance with the right to perform clinical tasks independently ( ten Cate, 2016). As such, preceptor observations and “subjective” judgments of a trainee’s ability provide a critical foundation for making trust decisions. In a study of decision variables in the entrustment process, the two highest ranked decision variables related to a learner’s demonstrated ability ( Duijn *et al*. 2018). According to Hauer *et al*. (2014), the building of a trust relationship is dependent upon the individuals, their shared relationship, the specific task being performed and the context for performance. ten Cate (2016) subsequently developed a framework for trust and suggested that for a supervisor to be willing to assume the risk associated with entrustment, four conditions must be met. First, the supervisor must determine that the trainee has the ability or competence to perform the task. Secondly, the supervisor must trust that the trainee will act with integrity and honesty. Thirdly, the trainee must be seen as reliable and conscientious in performing work duties and finally the trainee must act with humility and show a willingness to ask for help when needed. Our understanding of the contributions of each these four conditions to ad-hoc entrustment decisions remains unclear, however, all four elements are seen as important in decisions about readiness for indirect or unsupervised practice and promotion ( ten Cate 2016).

The concept of entrustable professional activities (EPAs) was more recently introduced to link competencies to clinical practice ( Rekman *et al*., 2016a; ten Cate, 2005; ten Cate, Snell and Carraccio, 2010) and trust decisions to workplace-based assessments that can better predict physicians’ actual performance ( Crossley and Jolly, 2012). Defined as tasks essential to professional practice, EPAs overcame the limitations of evaluating expected physician competencies in favor of a more direct approach of assessing what physicians actually do in daily practice ( ten Cate, Snell and Carraccio, 2010; ten Cate, 2013, Crossley and Jolly, 2012). As EPAs were expanded across specialties and subsequently adapted for undergraduate medical education (UME) to assess a student’s readiness for residency ( American Association of Colleges of Osteopathic Medicine or AACOM, 2016; Association of American Medical Colleges or AAMC, 2014; Basehore *et al*., 2017; Lomis *et al*., 2016), they became a unifying strategy for linking competencies to clinical practice at every level of medical training ( ten Cate, Snell and Carraccio, 2010; ten Cate 2013a). As such, the need for workplace-based assessments appropriate for capturing ad-hoc entrustment decisions for different levels of learners became paramount. Standard supervisory and co-activity EPA rating scales were established and later expanded to include levels of entrustment appropriate for medical students (Chen, van den Broek and ten Cate, 2015; Chen *et al*., 2016, Rekman *et al*., 2016b). The various scales now offer a standardized approach for measuring the entrustment of clinical duties to learners who are largely limited to practice under supervision.

The assessment of EPAs requires ad-hoc entrustment decisions by multiple raters who observe trainees in various contexts to produce reliable and generalizable data on which summative decisions about a learner’s overall readiness for unsupervised practice can be made ( Gruppen *et al*., 2018; Peters *et al*., 2017). For medical students, core clerkships provide an important opportunity to examine EPA ad-hoc entrustment decisions across a range of clinical contexts by multiple preceptors over time, and at the same time these clinical experiences provide the opportunity to explore the relationship between a student’s clinical ability, one of ten Cate’s (2016) four key conditions of trust, and the level of entrustment offered by the supervisor.

### Research questions

Ad-hoc entrustment decision relies, in part, on two factors: a) the trainees’ ability, in the case of this study, third-year medical students’ workplace-based performance on clerkships ( ten Cate, 2016), and b) the characteristics of specific clinical contexts, in the case of this study, the various clinical settings and patient encounters on different clerkships ( Hauer *et al*. 2014). The purpose of this study was to advance our understanding of the relationship between preceptors’ evaluation of students’ clinical ability, and ad-hoc entrustment decisions across a range of clinical contexts at the UME level. The specific research questions were:

• Given the varied clinical contexts of each clerkship, how is the entrustment decision for each EPA correlated with preceptor rating of a trainees’ ability on related clinical skills by clerkship?
• How is a specific entrustment level for an EPA linked to a specific preceptor rating of related clinical skills?

For the first research question, it was hypothesized that the level of entrustment would be positively correlated with the clinical performance ratings and that these correlations would vary by clinical contexts (clerkships). For the second research question, it was hypothesized that there would be consistency between preceptors’ level of entrustment and their ratings of clinical skills performance on clerkships. The outcomes of this study were intended to provide insight into the understanding of preceptors’ entrustment of students, and serve as a foundation for refining expected levels of performance by medical students across various clinical contexts.

## Methods

### Study Cohort

A retrospective study was conducted using a non-experimental design to assess the relationship between medical student clinical skills performance and preceptor entrustment decision. A total of 353 third-year medical students (Mean age= 23.6, 45% female, 15% underrepresented minority) who completed their core clerkships between June 2017 and June 2019 were included in the study.

### Instrumentation

Preceptor evaluation of clinical skills and their EPA entrustment decisions were collected using a workplace-based evaluation form during each of the seven core clerkships including Family Medicine, Internal Medicine, Pediatrics, Geriatrics, Psychiatry, Obstetrics/Gynecology (OB/GYN), and Surgery. This workplace evaluation form combined two existing instruments: 1) a workplace clinical skills evaluation scale, and 2) Chen’s supervisory scale for EPA entrustment ( Chen *et al*. 2016, Table 2).

### Clinical Skills Evaluation Scale

The workplace-based clinical skills evaluation scale was used to determine clerkship preceptor grades and was designed to assess specific knowledge, behaviors and skills within the six competency domains (medical knowledge, patient care, practice-based learning and improvement, interpersonal and communication skills, professionalism and systems-based practice), at a level appropriate for third-year medical students. The instrument included 15 items, each rated on a six-point scale ( *1=Unacceptable, 2= Developing, 3=Approaching competent, 4=Competent, 5= Approaching advanced, and 6=Advanced*), with behavior anchors specific to each clinical skill at four of the six points ( *Approaching competent* and *Competent* shared one behavioral anchor, as well as *Approaching advanced* and *Advanced*).

The current study linked specific clinical skills and underlying competencies on the evaluation form with EPAs, similar to the way in which an EPA supervisory scale has been used to align with competency milestone assessment ( ten Cate, 2016). Among the 15 items on the WBA, eight represented clinical behaviors or skills that were directly related to seven EPAs ( Table 1). The behavioral anchors for these eight skill items were likewise aligned with the specific functions described for each corresponding EPA (AACOM, 2016; AAMC, 2014). These eight skill items and the seven EPAs were used to evaluate the research questions in this study.

**Table 1.** Seven EPAs and Their Related Clinical Skills Items on the Workplace Evaluation Form

EPAs (5-level supervisory scale)	Related Clinical Skills Items (6-point rating scale)
EPA1. Gather a history and perform a physical examination	Q2. Takes an effective history
	Q3. Performs appropriate physical exam
EPA2. Prioritize a differential diagnosis following a clinical encounter	Q4. Generates a differential diagnosis that reflects clinical reasoning
EPA3. Recommend and interpret common diagnostic and screening tests	Q5. Recommends and interprets screening and diagnostic tests
EPA5. Document a clinical encounter in the patient record	Q9. Effective written documentation
EPA6. Provide an oral presentation of a clinical encounter	Q10. Effective oral presentation skills
EPA7. Form clinical questions and retrieve evidence to advance patient care	Q7. Demonstrates skills in evidence-based medicine
EPA9. Collaborate as a member of an inter-professional team	Q11. Teamwork skills

### Supervisory (EPA entrustment) Scale

The Chen, van den Broek and ten Cate (2015) entrustment scale ( Table 2) and 13 EPAs were added to the end of the existing workplace based evaluation form as a formative assessment that did not contribute to the student grade. The entrustment scale was scored from level 1 ( *not trusted to perform at all)* to level 5 ( *tr* *usted to perform unsupervised),* with an option to select *Unable to Evaluate* which was scored as a missing value. Preceptors were required to answer two questions before completing the entrustment scale: 1) *did they directly observe the student in patient care duties (Y/N)* and 2) *did they have meaningful interaction with the student and feel comfortable making entrustment decisions (Y/N).*The preceptor completed the level of entrustment for each EPA, as appropriate, based upon the following question, *Based upon your direct observation of the student, at what levelwould you entrust the student to perform the following skills during their next patient encounter.* The addition of the entrustment scale and EPAs to the preceptor form was reviewed and approved by the Clerkship Curriculum Committee.

**Table 2.** Entrustment (Supervisory) Scale

Level	Supervision Required to Perform EPA
1 2 3 4 5	Not trusted to perform at all Trusted to perform with direct (in-room) supervision only Trusted to perform with indirect supervision and all findings double checked Trusted to perform with indirect supervision and only key findings double checked Trusted to perform unsupervised

### Data collection

The clinical contexts of these core clerkships include both inpatient and outpatient settings, at different clinical sites. The workplace evaluation form was sent out to preceptors by the clerkship office through an online platform. Preceptors could opt to access and complete the form on a computer or mobile device. A student could be evaluated by multiple preceptors on a given clerkship, however only data from preceptors who had direct observations of and meaningful interactions with the students were included in the analysis. A total of 4217 forms were collected for 353 students, for an average of 12 (SD= 4) forms per student, an average of 602 forms (SD= 241) per clerkship, and 3373 forms (SD= 558) per EPA.

## Results/Analysis

The analyses focused on the seven EPAs (EPA 1, 2, 3, 5, 6, 7, and 9) and the eight corresponding clinical skills ( Table 1). For the first research question, a series of bivariate correlations between the pairs of EPA and clinical skills by clerkship were conducted. Because students received multiple preceptor evaluations within one clerkship, the ratings of their clinical skills and granted entrustment levels were averaged over multiple preceptors to obtain an aggregated score within each clerkship and across all clerkships. Then, Pearson’s correlations between average levels of entrustment in each of the seven EPAs and the average ratings of their linked clinical skills were examined across and within clerkships ( Table 3). With bonferroni adjustment (α= 0.05/64= 0.00078), all the correlations between the seven EPAs and their related clinical skills were statistically significant and positive across and within clerkships ( Table 3). In an analysis of data across all clerkships, there was a strong, positive correlations (r= 0.70-0.77) between entrustment of EPAs and the clinical performance rating for that skill. The strongest relationships were found between EPA2 entrustment and differential diagnosis skills performance rating (r= 0.77), followed by EPA1 with physical exam performance (r= 0.76), and EPA6 with oral presentation skills performance (r= 0.76).

Table 3 also showed varied correlations between entrustment levels and clinical skills ratings by clerkships. The strongest correlations were found among Family Medicine (r= 0.63-0.75), Surgery (r= 0.61- 0.74) and Geriatrics (r= 0.55-0.70). Although statistically significant, Obstetrics and Gynecology (r= 0.39-0.51) and Psychiatry (r= 0.43-0.55), by contrast, reflected consistently weaker relationships across all EPAs and clinical skills. Overall, the relationship between clinical performance and entrustment level ranged from moderate to strong, and varied by EPAs and clerkships. In addition, these positive correlations indicate that the stronger a student performed on a specific skill in the workplace (higher skill ratings), the higher was their level of entrustment for that same skill.

**Table 3.** Correlations between EPA Entrustment Level and Preceptor Rating of Clinical Skills across and within Clerkships

		EPA 1 With		EPA 2 with	EPA3 With	EPA5 with	EPA6 With	EPA7 with	EPA9 with
		Clinical Skills Item(s) on Workplace Evaluation Form							
Clerkship	Weeks	History Taking	Physical Exam	Differential Diagnosis	Screening and Diagnostic Tests	Document-ation	Oral Presentation	Evidence-based Medicine	Teamwork
*Across Clerkships*		*0.75* *	*0.76* *	*0.77* *	*0.75* *	*0.70* *	*0.76* *	*0.72* *	*0.75* *
Family Medicine	8	0.63 *	0.63 *	0.69 *	0.75 *	0.66 *	0.65 *	0.71 *	0.66 *
Geriatrics	4	0.67 *	0.69 *	0.61 *	0.55 *	0.61 *	0.67 *	0.70 *	0.66 *
Internal Medicine	6	0.54 *	0.58 *	0.67 *	0.59 *	0.50 *	0.60 *	0.56 *	0.59 *
OB/GYN	4	0.46 *	0.51 *	0.40 *	0.49 *	0.39 *	0.44 *	0.40 *	0.50 *
Pediatrics	4	0.61 *	0.60 *	0.61 *	0.59 *	0.50 *	0.56 *	0.47 *	0.58 *
Psychiatry	4	0.47 *	0.53 *	0.51 *	0.55 *	0.46 *	0.46 *	0.43 *	0.53 *
Surgery	6	0.66 *	0.67 *	0.61 *	0.63 *	0.63 *	0.63 *	0.64 *	0.74 *

* indicates significant at p< 0.00078, after bonferroni adjustment

**Figure 1.**
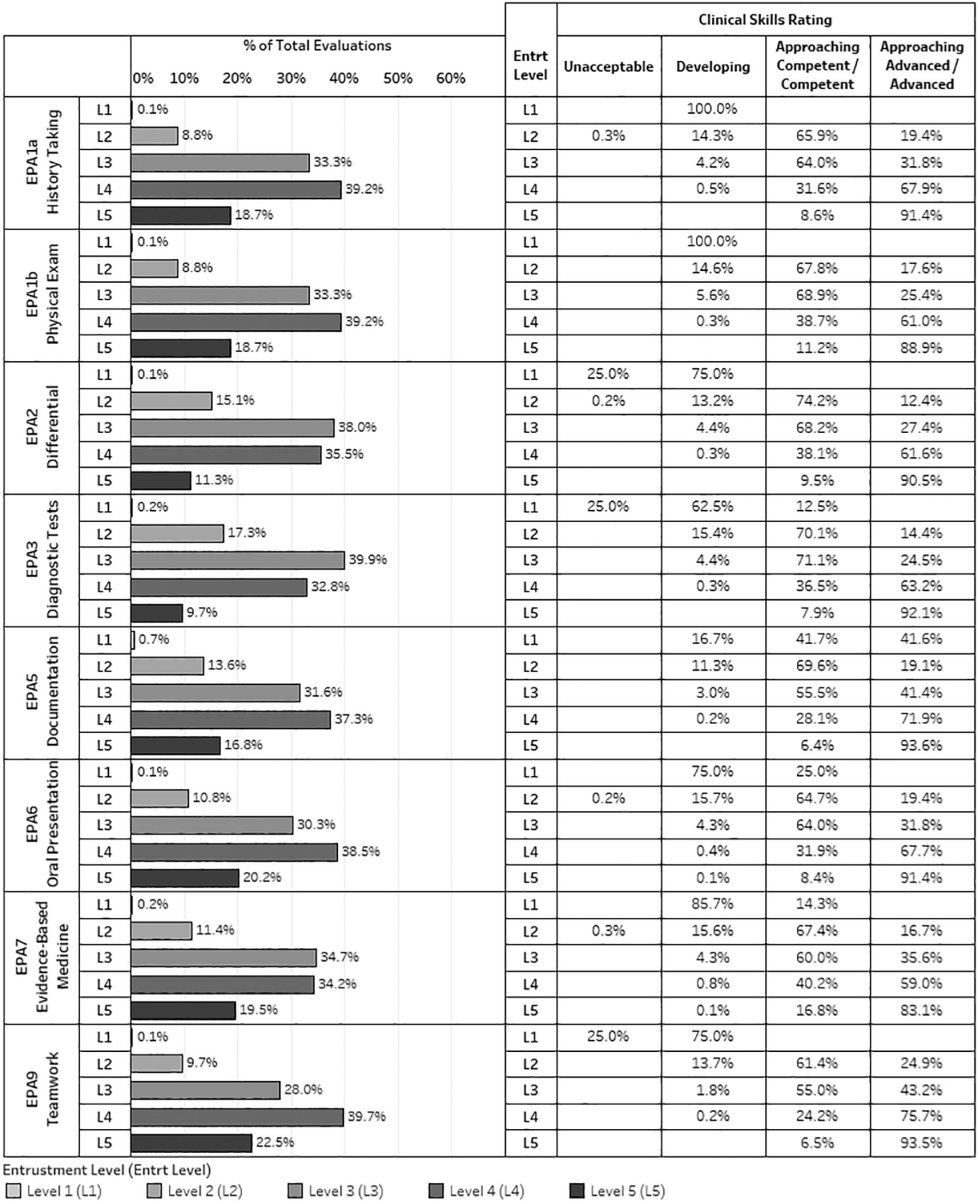
The distribution of entrustment level by each EPA (the left bar charts), and the distribution of corresponding clinical skills ratings by each entrustment level (the right stacked table)

The second research question explored the relationship between the level of entrustment for each EPA and the specific preceptor rating for the related clinical skill. Unlike the first research question, the unit of analysis for this research question was each evaluation instead of each student, therefore the raw levels and raw ratings were used, instead of averages. Simple crosstabs of the seven EPAs and their corresponding clinical skills were used to examine the patterns of entrustment. Since the ratings of *approaching competent and competent* shared a common behavior anchor, the two ratings were collapsed into one category for the purpose of this analysis, as was *approaching advanced and advanced.* Figure 1 depicts the distribution in the levels of entrustment by EPA (bar charts) and the corresponding clinical skill performance rating level on the preceptor rating scale (stacked table). For example, for EPA 1a ( *history taking*), the level 1 entrustment ( *not trusted to perform at all*) was consistently associated with a preceptor rating of *developing* on the evaluation of clinical performance for that related skill. That is to say, for students granted with level 1 entrustment on EPA 1a, 100% of them were rated by their preceptor as *developing* in terms of their demonstrated history taking skill.

The distribution in the levels of entrustment for each of the seven EPAs showed that preceptors most frequently granted level 3 ( *trusted to perform with indirect supervision and all findings double checked*) and level 4 ( *trusted to perform with indirect supervision and only key findings double checked*) entrustment. The percentage of all entrustment decisions at level 3 or 4 ranged from 67.7% -73.5% across EPAs. Level 1 entrustment ( *not trusted to perform at all*) was consistently linked with a clinical skill performance rating of *developing* (from 62.5% in EPA3 to 100% in EPA1) for all EPA except EPA 5 (written documentation), while the highest level of entrustment ( *trusted to perform unsupervised*) was primarily linked with a clinical skill performance rating of *approaching advanced/advanced (*from 83.1% in EPA7 to 93.6% in EPA5 *).* However, for the three levels of entrustment in the middle (levels 2-4), the link between individual entrustment level and a particular clinical skill performance rating was less salient. Clinical skill performance ratings associated with these three levels of entrustment varied, particularly for entrustment levels 3 and 4. For example, among those granted level 3 entrustment on EPA5, a small majority (55.5%) were rated *approaching competent/competent* on their documentation skills, while a substantial percentage (41.4%) were also rated *approaching advanced/advanced.* Similar patterns of skill ratings were seen for entrustment level 4 for EPA7 and level 3 for EPA9.

Overall, Figure 1 indicated that the pattern of association between individual entrustment level and skill rating varied by EPAs as well as level of entrustment. Regarding the difference in level of entrustment, there was less ambiguity at the lowest and highest levels of entrustment across all seven EPAs, primarily linked with the clinical skill ratings of *Developing* and *Approaching advanced/ Advanced*, respectively. There were likewise differences across EPAs. The pattern of association for EPAs 1 and 2 was more salient than that for EPAs 5, 7 and 9.

## Discussion

There are many factors that can influence the trust a preceptor places in a trainee, not the least of which is the student’s prior years of training and experience. Medical students work under supervision often with limited autonomy to perform clinical skills given regulatory constraints and concern for patient safety. As such, differing levels of entrustment have been proposed for different stages of medical training. Chen’s supervisory scale used in this study offers more gradations of supervision to allow for more gradual development of autonomy among medical students. Trusted to perform with *indirect supervision* and *key findings double checked*, has been suggested as the appropriate level for transition from student to resident (Chen, van den Broek and ten Cate, 2015). Still others ( Hodges, 2013) have suggested a simpler dichotomous determination of those not ready for indirect supervision (pre-entrustable) and those who are (entrustable), although one study on the use of clinic EPA assessment cards on a surgical clerkship found the binary scale not useful ( Curran *et al.*, 2018). Given that the supervisory scale provides greater detail about the level of supervision required to entrust EPAs to medical students, it serves as an appropriate scale to explore the research questions in the current study.

The findings of this study added empirical evidence to our understanding of ad-hoc entrustment decisions across varied clerkship contexts and the relationship of these decisions to a trainee’s ability, one of ten Cate’s (2016) general conditions of trust. Previous literature that explored the relationship of students’ clinical ability and entrustment has been limited to the context of clinical simulations or objective structured clinical exams (OSCEs) ( Eliasz *et al*., 2018; Holzhause, *et al.*, 2019). This current study filled the gap by providing insights into the conditions of trust in real clinical workplace. The moderate to strong positive correlations between the seven undergraduate EPAs and their performance ratings on the workplace based evaluation showed that trust in the clinical setting was to some extent dependent on the trainees’ demonstration of their clinical skills or competencies. Also, the positive correlations confirmed ten Cate’s assumption (2016) that the specific level of supervision decreases (higher levels of entrustment), as students increase in their competence and clinical skills.

The expected level of entrustment is often dependent on the clinical context and the specific EPA skill, and can also be influenced by the subjective nature of rater decisions ( ten Cate, 2013a). Results of this study showed the relationship between entrustment and clinical skill performance varied by EPAs as well as by clerkship, consistent with the suggestion that context impacts the establishment of trust and therefore entrustment decisions ( Hauer *et al.*, 2014). The continuity in clinical training experiences and mentorship are two characteristics known to be conducive for developing trust relationships (Hirsh, Holmboe and ten Cate, 2014). Consistent with these findings, this study documented its strongest correlation between performance and entrustment on the Family Medicine clerkship. This 8-week clerkship is our longest and one in which the students worked in the outpatient setting with close supervision by just a few preceptors, unlike most other clerkships such as psychiatry, obstetrics/gynecology and pediatrics, where students worked in the hospital setting with rotating preceptors for a much shorter period of time. Thus, in the other clerkships, there were lower associations between EPA entrustment and student clinical performance. For the three disciplines, students were also less likely to be trusted with common tasks such as data gathering (history taking and physical examination) and documentation. This finding likely reflects the unique skills required to care for these specific patient populations and the additional supervised training required during clerkship.

Some of the differences in EPA entrustment were consistent across clerkships. For example, the correlation between EPA entrustment and clinical performance was more modest for EPA 5 (written documentation) and EPA 6 (forming clinical question and retrieving evidence) across clerkships. Both skills were less likely to be directly observed and therefore more likely to be inferred from student oral presentations leading to the potential for a misalignment between performance rating and entrustment. The use of proxy data over direct observation to assess clinical skills is less likely to generate reliable ratings of clinical performance and is not supported by the evidence ( Kogan *et al*., 2017). History and physical examination skills and oral presentation skills by contrast had stronger associations between performance ratings and entrustment for most clerkships. These skills are introduced early in student training and practiced extensively in simulation and case-based learning activities throughout the pre-clerkship years. Given this early exposure, students generally demonstrate greater ability and independence in performing these skills, have more of an opportunity to demonstrate the skill on rotation and therefore are more likely to be trusted to function with more limited supervision.

The current study further advanced our understanding of the relationship between entrustment decisions and a trainee’s clinical ability by exploring the patterns between specific entrustment level and clinical performance ratings on related skills. Results in Figure 1 showed overall students were most frequently trusted with *indirect supervision* when they demonstrated *approaching competent* to *competent* level performance, and *without supervision* when they demonstrated *approaching advanced* to *advanced* level of performance across EPAs. But, such patterns also varied by EPA. For EPAs that students had more of an opportunity to practice (e.g. EPA1 or 2), the connections are more salient (a specific entrustment level was predominantly associated with a specific skill rating, e.g. level 1 entrustment on history taking and physical examination (EPA1) was 100% linked with a performance rating of *developing.* This could be due to having more reliable evaluations for these commonly demonstrated skills on rotation (e.g. 4002 evaluations for EPA2 versus 3209 evaluations for EPA5), or that preceptors were more likely to have established expectations for frequently observed skills and greater consistency in relating entrustment decision to specific rating of trainees’ clinical skills for these EPAs. The patterns observed between the levels of entrustment and specific skill ratings provided important insight about preceptors’ expectations of student performance across various clinical contexts. The findings from this study can help guide undergraduate medical educators better prepare students to become resident ready as they advance in training.

A limitation of the present study is that it provided evidence on only one of the four conditions suggested to influence trust decisions, the relationship between ad-hoc entrustment decisions and clinical skill performance or ability. In practice, other factors such as integrity, reliability and humility, may be needed for responsible decisions of students’ readiness for higher levels (indirect or unsupervised) of supervision ( ten Cate, 2016). Additionally, the ad-hoc entrustment decisions were captured as part of an end-of-clerkship assessment and therefore did not capture specific differences in individual experiences such as patient complexity. Given the evidence of varied relationships between different EPAs and their related clinical skills, it may suggest the need to use discrete supervisory scales for each EPA to capture context details important to entrustment decisions unique to each EPA. The more granular approach to capturing the contexts for entrustment decisions will better support summative decisions about a student’s readiness for residency and unsupervised practice, as well as support faculty development on entrustment decisions.

## Conclusion

The study explored the relationship between a medical student’s clinical ability and the ad-hoc entrustment offered by preceptors in clinical settings across a range of contexts on third year clerkships. The findings support the relationship between students’ performance and entrustment decisions. Stronger clinical performance elicited greater entrustment from preceptors, although the level of performance associated with specific levels of entrustment varied by clerkship and EPA. Clerkships provide an effective environment for capturing performance across a range of patient care contexts, although discrete forms to collect more granular information about individual ad-hoc entrustment decisions such as patient care complexity, as well as learner characteristics (e.g. integrity, humility) may prove to be more effective than end-of-rotation assessments.

## Take Home Messages

• There is need for more empirical research on the relationship between a learner’s clinical ability as a general condition of trust and ad-hoc entrustment decisions.
• Clerkships can be used to capture ad-hoc entrustment decisions across a range of clinical contexts and examine the relationship between student performance and the level of entrustment for EPA skills. Longer clerkships with more individualized mentorship provide important opportunities for preceptors to meaningfully build trust and make EPA entrustment decisions.
• Stronger clinical performance elicited greater entrustment from preceptors, although the level of performance associated with specific levels of entrustment varied by clerkship and EPA.
• Discrete entrustment tools designed to collect more detailed information about context, such as patient care complexity, as well as learner characteristics (integrity, humility) may prove to be more effective than end-of-clerkship assessments at capturing ad-hoc entrustments that support summative assessment of a learner’s readiness for unsupervised practice.

## Notes On Contributors

**Pamela Basehore**, EdD, MPH, is an Associate Professor at the Department of Geriatrics and Gerontology and Associate Dean for Assessment and Evaluation at Rowan University School of Osteopathic Medicine, Stratford, New Jersey, USA.

**Shiyuan Wang**, PhD, is an Assistant Professor at the Department of Geriatrics and Gerontology and Assessment Specialist for Office of Assessment and Evaluation at the Rowan University School of Osteopathic Medicine, Stratford, New Jersey, USA.

**Mehwish Khan**, is a second-year medical student at Rowan University School of Osteopathic Medicine, Stratford, New Jersey, USA.

## Declarations

The author has declared that there are no conflicts of interest.

## Ethics Statement

The study was approved on 17th January 2018 by Rowan University School of Osteopathic Medicine Institutional Review Board, approval no. Pro2017002108.

## External Funding

This is partially funded by American Association of Colleges of Osteopathic Medicine Medical Education Research Grant.
