# Supplementary material for: Relationship of Preceptor Ad-hoc Entrustment Decisions to Students’ Clinical Skills Performance
Source: MedEdPublish (2016). 2020 Apr 2;9:61. [Version 1] doi: 10.15694/mep.2020.000061.1 (PMC10697435; doi:10.15694/mep.2020.000061.1)
Supplement: Peer review history

# Reviewer report: Reviewer response for version 1

**DOI:** 10.21956/mep.20031.r30606

**Published:** 2020-05-28

**Authors:** Ken Masters

**Affiliations:** 1 Sultan Qaboos University

This review has been migrated. The reviewer awarded 3 stars out of 5 An interesting study on the relationship of preceptor ad-hoc entrustment decisions to students’ clinical skills performance. Overall, a strong piece of research, bearing in mind the queries and limitations mentioned by the other reviewers. The researchers have laid out the needs and aims clearly, and have presented the results in a manner that is easy to read.Two points regarding the sample’s demographics:• On reporting demographics of the sample, such as gender and minority, it is best to report on all statistics, in order to reduce the risk of accusations of prejudice or inappropriate assumptions (or lead to incorrect assumptions by your readers). In the case of a minority, as this will differ vastly from country to country, and MedEdPublish is an international journal, it would be best to more specifically identify or define the classification.• Related to this, the significance of determining the demographics is not clear. The information is gathered, presented, and then not mentioned again. If these variables were never to be tested against the other data, then, (apart from a general description) the reason for gathering them in the first place is unclear. Given that there were variations across the findings, an exploration in relation to the demographics may assist in clarity (and, if there are no significant relationships, then, at least, that would allow one to exclude those as influencing factors).I look forward to a Version 2 of the paper in which these and the other reviewers’ points are addressed.

Reviewer Expertise:

NA

No decision status is available

# Reviewer report: Reviewer response for version 1

**DOI:** 10.21956/mep.20031.r30605

**Published:** 2020-04-08

**Authors:** Julie Hunt

**Affiliations:** 1 Lincoln Memorial University

This review has been migrated. The reviewer awarded 4 stars out of 5 This research paper investigates what a reader may think is an intuitive link between strong clinical skills and an increased likelihood of an entrustment decision being made. The researchers lay out a strong background of literature in the introduction, which is one of the paper's strongest points, setting the stage for the data to be collected. Another of the paper's strength is the quantity of data collected, which does support the intuitive link between clinical skills and entrustment decisions. There was some information missing from the methods. For example, who did the ratings, and when? Later in the discussion it is revealed that the entrustment ratings were made at the end of each rotation, but the reader is never told who is doing the ratings, or whether it is even always the same person (i.e. is pediatrics always scored by the same rater?)The results point out that the link is weakest in the OB/gyn and psychiatry services, and the researchers partially explain this by these rotations being shorter in duration and less accepting of students performing even common tasks such as data gathering and documentation. Since the issue of duration of rotation was brought up, I would have liked to have seen that data presented in the results section. How long was each rotation, and how did that impact the correlation coefficient, numerically speaking? I would also have liked to have seen a more complete discussion of study limitations, including natural variability in raters, use of a single institution and cohort, etc.

Reviewer Expertise:

NA

No decision status is available

# Reviewer report: Reviewer response for version 1

**DOI:** 10.21956/mep.20031.r30604

**Published:** 2020-04-03

**Authors:** Deb Halder

This review has been migrated. The reviewer awarded 5 stars out of 5 Notes on Abstract, Introduction and References:This research paper tends to study on the two variables: preceptor ad-hoc entrustment and student’s clinical skills performance that fosters relationship. As per the concepts of research paper, the abstract discussed the complete design of the paper. The abstract designates what to study and how to study. The introductory part evaluated the key conceptions of the paper which defended with reasonable, updated references. The literature reviews makes this paper strong in its assumption. The delineation of research questions and hypothesis clarified the objective of the paper.Methodology:The methodology is detailed in expression which tends to be reliable and generally adoptable. Analysis and Discussion:The analysis presented data with tables. Few more graphs would have been acceptable to have the data more visibility and transparent. The long all informative graph could have been divided with parts along with its analysis.The discussion is well articulated and has made a comparative study with the previously cited references; this claims praise. It needs to be suggested that the limitation should have taken a different sub-title for better readability; along with the limitation, delimiting process may be addressed though not mandatory for this paper.Concluding remarks:The culminating part is whole in its scope and quality. This is well deliberated thought and study presented by the authors. This paper is to be praised for its content and construction.

Reviewer Expertise:

NA

No decision status is available

# Reviewer report: Reviewer response for version 1

**DOI:** 10.21956/mep.20031.r30607

**Published:** 2020-04-02

**Authors:** Felix Silwimba

**Affiliations:** 1 University of Lusaka

This review has been migrated. The reviewer awarded 5 stars out of 5 informative and educational report . on competence evaluation of medical students.

Reviewer Expertise:

NA

No decision status is available
